# Paradoxical relationship between body mass index and bone mineral density in patients with non–small cell lung cancer with brain metastasis

**DOI:** 10.1371/journal.pone.0218825

**Published:** 2019-06-21

**Authors:** Min Woo Nam, Jae Min Kim, Jin Hwan Cheong, Je Il Ryu, Myung-Hoon Han

**Affiliations:** Department of Neurosurgery, Hanyang University Guri Hospital, Guri, Gyonggi-do, Korea; Seoul National University College of Pharmacy, REPUBLIC OF KOREA

## Abstract

**Background and purpose:**

Low body mass index (BMI) at presentation has been reported to be associated with higher incidence and mortality of lung cancer, but studies on the relationship between brain metastasis and BMI at presentation are lacking. This study aimed to evaluate the association between brain metastasis and BMI and bone mineral density (BMD) in NSCLC.

**Methods:**

We retrospectively enrolled patients with non–small cell lung cancer who underwent brain magnetic resonance imaging with contrast within 3 months of diagnosis. The BMI was collected, and the BMD was measured in Hounsfield unit (HU) on initial staging computed tomography scans. The independent relationship between BMI and BMD was assessed using multivariable linear regression according to the presence of brain metastasis.

**Results:**

A total of 356 consecutive NSCLC patients were enrolled in the study over a 8-year period in a single institution. Lower BMI with higher BMD was an independent predictive factor for brain metastasis in patients with NSCLC, relative to the other group (HR, 2.03; 95% CI, 1.21 to 3.40; P = 0.007). We also found a significant negative correlation between BMI and BMD among patients with NSCLC with brain metastases (B, -3.343; 95% confidence interval, -6.352 to -0.333; P = 0.030).

**Conclusions:**

Brain metastasis may possibly be associated with lower BMI and higher BMD in NSCLC patients. We expect that these results may facilitate future predictions of brain metastases during the clinical course of NSCLC and enhance our understanding of the underlying mechanisms that link brain metastases and lung cancer.

## Introduction

Patients with lung cancer often have stage IV disease and distant metastasis on initial diagnosis; lung cancer metastasizes via both the lymphatics and blood vessels [1]. Brain metastases are the most common intracranial complications of cancer in adults, and non–small cell lung cancer (NSCLC) is among the most common primary tumor to develop brain metastasis [2]. Brain metastasis is a devastating complication of systemic malignancy and generally shows poor prognosis with shorter survival time. Systemic therapies may improve survival with lack of intracranial penetration, there will be more chance to develop brain metastases [3].

Previous studies [3–8] reported that the common clinical risk factors for brain metastases in NSCLC patients include younger age, female sex, adenocarcinoma cell histology, and higher tumor-node-metastasis (TNM) stage with emphasis on lymph node involvement. In addition, initial lower body mass index (BMI) has been widely reported to be associated with higher mortality and incidence of lung cancer [9–12]. However, studies on the relationship between brain metastasis and BMI at initial diagnosis are lacking. BMI is known to be associated with bone mineral density (BMD), and thus BMD, which is measured in Hounsfield unit (HU) in L1, is also commonly assessed during diagnosis for other indications [13,14]. BMD is generally assessed via computed tomography (CT) because HU values obtained on regional CT scans is significantly correlated with the true BMD [13,15,16].

This study aimed to examine the relation between brain metastasis and BMI and BMD in NSCLC patients.

## Methods

### Patient selection

This study was approved by the Institutional Review Board of Hanyang University Guri Hospital, Korea, and conformed to the tenets of the Declaration of Helsinki. Owing to the retrospective nature of the study, the need for informed consent was waived. All patient records were anonymized prior to analysis.

Among all patients who visited or were admitted to Hanyang University Guri Hospital from January 1, 2007 to December 31, 2014, we retrospectively recruited patients with primary lung cancer who underwent brain magnetic resonance imaging (MRI) with contrast within 3 months of diagnosis. Patients (1) who underwent staging workup in another institution; (2) have incomplete initial staging workup (including pathological diagnosis); (3) have SCLC; (4) have missing data, including height and weight; and (5) have compression fracture or bone cement in the L1 vertebral body were excluded. We collected information on BMI at lung cancer diagnosis and measured HU values on initial staging CT scans.

### Staging workup

Initial staging workup was conducted by obtaining a detailed medical history, physical examination, and imaging, including chest radiography, contrast-enhanced CT of the chest and abdomen, brain MRI with contrast, whole-body bone scintigraphy, bronchoscopy, and positron emission tomography/CT. All radiological findings were confirmed by faculty radiologists. NSCLC was pathologically confirmed via biopsy. Most brain metastases were diagnosed based on the results of contrast-enhanced brain MRI because most patients were ineligible for surgery. Meanwhile, pathological diagnoses were established via surgical biopsy in patients with brain metastasis who were treated via surgical resection. The initial TNM stage at diagnosis, excluding brain metastasis, was determined according to the seventh edition of the Cancer Staging Manual of the American Joint Commission on Cancer [17].

### BMI classification

Body mass index (BMI) was calculated as weight/(height)^2^ and expressed in kg/m^2^. The patients’ weight and height at lung cancer diagnosis were collected from the medical records. BMI was classified according to the World Health Organization Asian BMI classification: <18.5 kg/m^2^, underweight; 18.5–22.9 kg/m^2^, normal; 23.0–27.5 kg/m^2^, overweight; and >27.5 kg/m^2^, obese [18].

### Assessment of HU values in the L1 vertebral body

**Fig 1:**
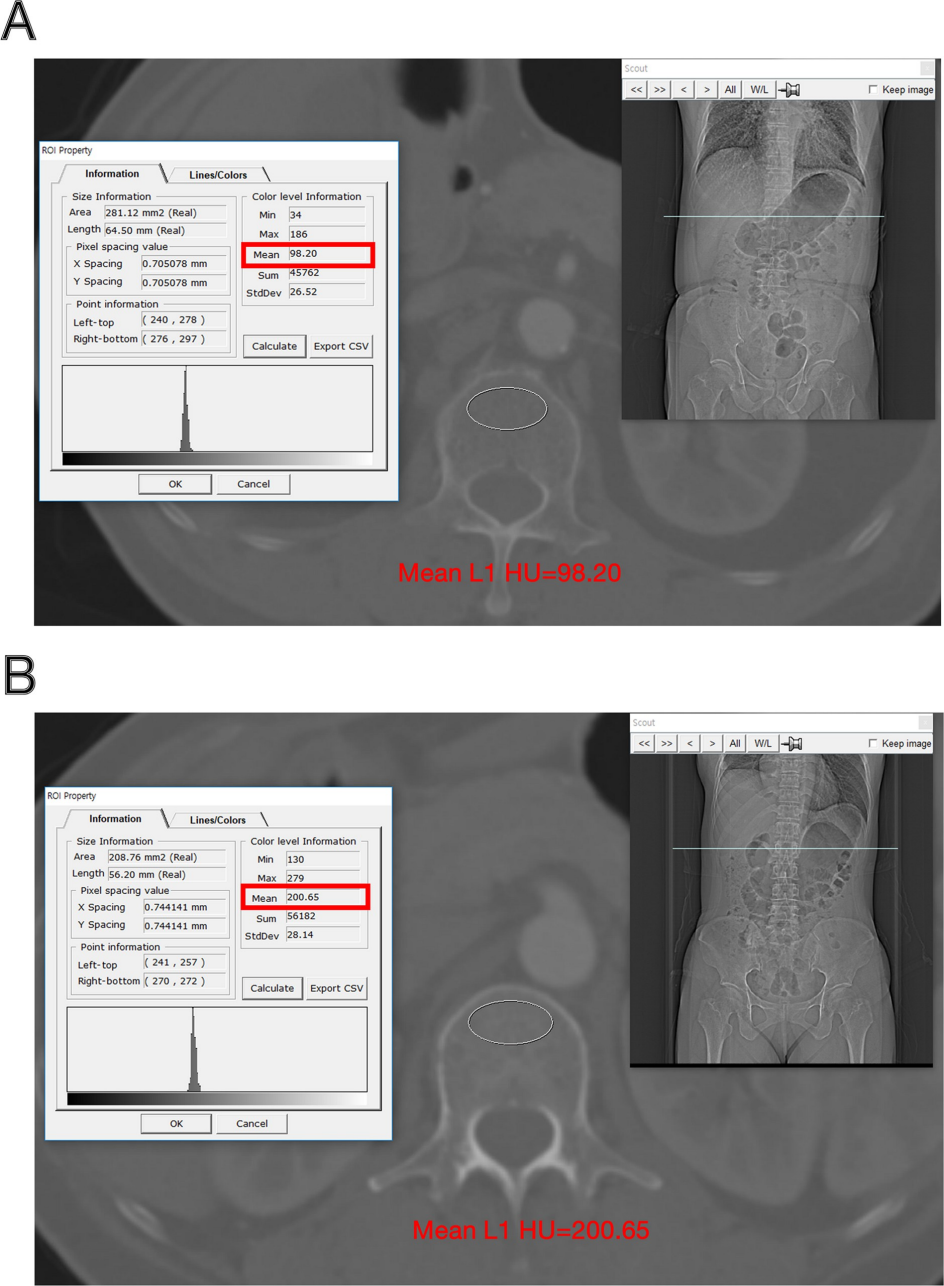
Examples of measurement of HU values in the trabecular bone of the middle of the L1 vertebral body. The PACS automatically calculates the mean HU value according to ROI line, and the mean HU value is recorded. (A) CT showing relatively low mean L1 HU value; (B) CT showing relatively high mean L1 HU value. HU = Hounsfield unit; PACS = picture archiving and communication system; ROI = region of interest.

All CT images were obtained with Siemens multidetector CT scanners and reviewed using the picture archiving and communication system. We measured the HU values in the trabecular bone of the middle of the L1 vertebral body from the chest and abdominal CT scans performed for the initial staging workup. All HU values were measured by a faculty neurosurgeon blinded to the clinical data with CT images that were magnified on the bone setting. HU value in the vertebral body was measured following the method of Pickhardt et al. (Fig 1) [13].

Based on their study, the L1 vertebral was also measured, and we followed the threshold (balanced sensitivity and specificity) of 160 of L1 HU for distinguishing normal from low BMD (osteopenia and osteoporosis). Because the HU value on CT is an accurate absolute value with validity and reproducibility ranging from 0 HU to 20 HU between all appropriately calibrated CT scanners [19,20].

### Other study variables

The electronic medical records of all patients were reviewed by two study staff. Surgical resection was performed in patients with early stage NSCLC. Systemic chemotherapy was administered with or without radiotherapy based on routine platinum-based two-drug combination with two to six cycles in the first-line treatment of advanced NSCLC [21]. Radiotherapy was delivered using a linear accelerator with a radiation dose ranging from 5000 to 6600 cGy (200 cGy per fraction in 25–33 fractions). Smoking history was also included in the analysis.

### Statistical analysis

To identify differences between patients with and without brain metastasis categorical and continuous variables were examined via the Chi square test and Student’s *t*-test, respectively. The time duration between lung cancer detection and brain metastasis was defined as the days between diagnosis of lung cancer on imaging and the first appearance of brain metastasis on brain MRI. According to a recent study from Korea, we set the end-point of study at three years (1095 days) from lung cancer detection [22]. The cumulative hazard for brain metastasis was examined using Kaplan-Meier analysis classified according to several predictive factors, with censoring of patients who had no brain metastasis on the last brain MRI. Hazard ratios (HRs) with 95% confidence intervals (CIs) were then calculated using uni- and multivariate Cox regression analysis to identify independent predictive factors for brain metastasis. Sex, age (continuous variable), BMI classification, L1 HU, histology, T stage, N stage, distant metastasis (other than in the brain), initial treatment, and smoking history in pack-years were entered into the multivariate model.

Multivariable linear regression analysis was also performed to identify the independent relationship of these covariates with L1 HU value according to the presence of brain metastasis. A *P* value <0.05 was considered statistically significant.

All statistical analyses were performed using R software version 3.5.1 (https://www.r-project.org/).

## Results

### Patients

Of the 555 consecutive patients initially identified, 199 were excluded because their staging workup was conducted in another institution (n = 22), have incomplete initial staging workup (n = 37), have SCLC (n = 104), have missing data (n = 29), and have compression fracture or bone cement in the L1 vertebral body (n = 7). Finally, 356 consecutive patients with NSCLC were enrolled in the study over an 8-year period. The average patient age was 69.3 years, and 25.8% of patients were female. We identified 103 patients who developed brain metastases from NSCLC, and mean period between lung cancer detection and the last brain MRI was 226.8 days. There were significant differences in sex, BMI, histological type, N stage, distant metastasis, and initial treatment between patients with and without brain metastasis (Table 1).

**Table 1:** Characteristics of the study patients according to brain metastasis.

Characteristics	Brain metastasis (-) (n = 253)	Brain metastasis (+) (n = 103)	Total (n = 356)	*P*
Female sex, n (%)	55 (21.7)	37 (35.9)	92 (25.8)	0.008
Age (years), mean ± SD	69.6 ± 9.6	68.8 ± 11.6	69.3 ± 10.2	0.551
Time duration between lung cancer diagnosis and the last brain MRI (days), mean ± SD	223.9 ± 509.9	234.2 ± 465.0	226.8 ± 496.8	0.861
Time duration between lung cancer diagnosis and brain metastasis development (days), mean ± SD	N/A	158.1 ± 440.7	N/A	N/A
BMI, mean ± SD, kg/m^2^	23.2 ± 3.8	22.3 ± 3.6	22.9 ± 3.7	0.045
BMI, median (IQR), kg/m^2^	23.4 (20.7–25.6)	22.4 (19.8–24.5)	23.1 (20.5–25.3)	0.045
BMI classification, n (%)				0.074
Underweight (<18.5 kg/m^2^)	25 (9.9)	18 (17.5)	43 (12.1)	
Normal (18.5–22.9 kg/m^2^)	89 (35.2)	42 (40.8)	131 (36.8)	
Overweight (23.0–27.5 kg/m^2^)	114 (45.1)	37 (35.9)	151 (42.4)	
Obese (>27.5 kg/m^2^)	25 (9.9)	6 (5.8)	31 (8.7)	
L1 HU value, median (IQR)	124.1 (93.7–157.2)	136.8 (95.8–175.8)	126.2 (94.2–160.1)	0.124
L1 HU value, mean ± SD	129.5 ± 52.2	139.1 ± 56.7	132.2 ± 53.7	0.124
Histological type, n (%)				<0.001
Adenocarcinoma	127 (50.2)	77 (74.8)	204 (57.3)	
SCC	112 (44.3)	17 (16.5)	129 (36.2)	
Others	14 (5.5)	9 (8.7)	23 (6.5)	
T stage, n (%)				0.051
T1	100 (39.5)	25 (24.3)	125 (35.1)	
T2	83 (32.8)	45 (43.7)	128 (36.0)	
T3	46 (18.2)	21 (20.4)	67 (18.8)	
T4	24 (9.5)	12 (11.7)	36 (10.1)	
N stage, n (%)				<0.001
N0	104 (41.1)	17 (16.5)	121 (34.0)	
N1	33 (13.0)	11 (10.7)	44 (12.4)	
N2	55 (21.7)	35 (34.0)	90 (25.3)	
N3	61 (24.1)	40 (38.8)	101 (28.4)	
Distant metastasis (other than in the brain), n (%)				0.048
M0	176 (69.6)	60 (58.3)	236 (66.3)	
M1	77 (30.4)	43 (41.7)	120 (33.7)	
Initial treatment, n (%)				0.001
Supportive care	85 (33.6)	44 (42.7)	129 (36.2)	
Surgery only	34 (13.4)	3 (2.9)	37 (10.4)	
Chemotherapy only	67 (26.5)	43 (41.7)	110 (30.9)	
Radiotherapy only	8 (3.2)	1 (1.0)	9 (2.5)	
Surgery + chemotherapy	33 (13.0)	5 (4.9)	38 (10.7)	
Surgery + radiotherapy	2 (0.8)	0 (0.0)	2 (0.6)	
Chemotherapy + radiotherapy	24 (9.5)	7 (6.8)	31 (8.7)	
Smoking status, n (%)				0.541
Never	112 (44.3)	52 (50.5)	164 (46.1)	
Former	56 (22.1)	19 (18.4)	75 (21.1)	
Current	85 (33.6)	32 (31.1)	117 (32.9)	
Smoking history in pack-years				0.088
Never	112 (44.3)	52 (50.5)	164 (46.1)	
<30	37 (14.6)	21 (20.4)	58 (16.3)	
30–49	58 (22.9)	21 (20.4)	79 (22.2)	
≥50	46 (18.2)	9 (8.7)	55 (15.4)	

NSCLC, non–small cell lung cancer; MRI, magnetic resonance imaging; SD, standard deviation; BMI, body mass index; IQR, interquartile range; HU, Hounsfield unit; SCC, squamous cell carcinoma; N/A, not available

S1 Table shows the detailed information on brain metastasis in the study cohort. The rate of multifocal metastasis (≥ 3 metastasis) was 47.6%, and the most frequent metastatic site were the frontal and parietal lobes. Four patients were suspected of brain metastasis first and subsequently diagnosed with primary NSCLC. For these patients, the time duration was recorded as 0 (Day 0).

### Cumulative hazard of brain metastasis according to BMI and BMD

**Fig 2:**
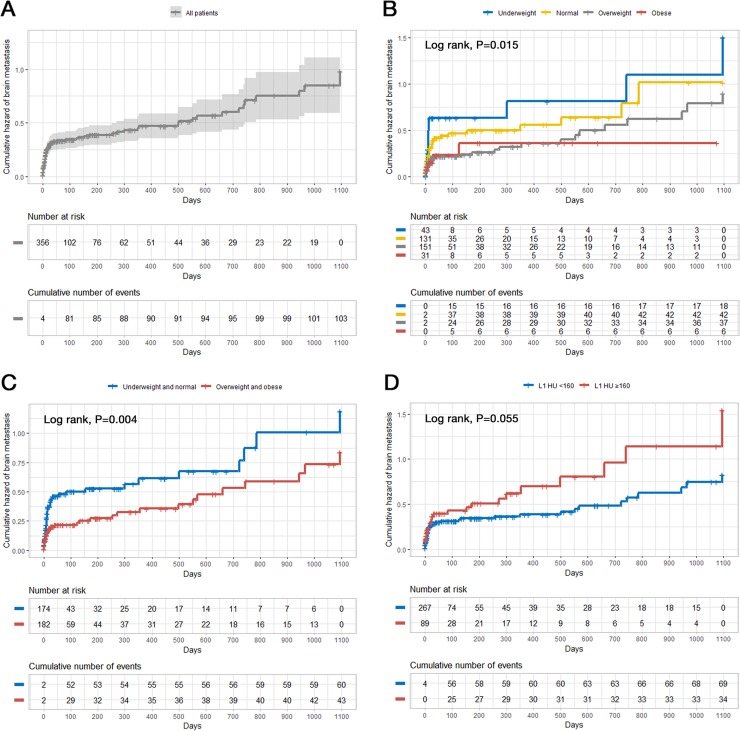
Cumulative hazard of brain metastasis according to the BMI and L1 HU classification. (A) overall; (B) four BMI categories (underweight, normal, overweight, and obese); (C) two BMI categories (underweight and normal, overweight and obese); (D) L1 HU classification (cut-off value of 160 HU). BMI = body mass index; HU = Hounsfield unit.

Fig 2A shows the overall cumulative hazard of brain metastasis after NSCLC diagnosis.

**Fig 3:**
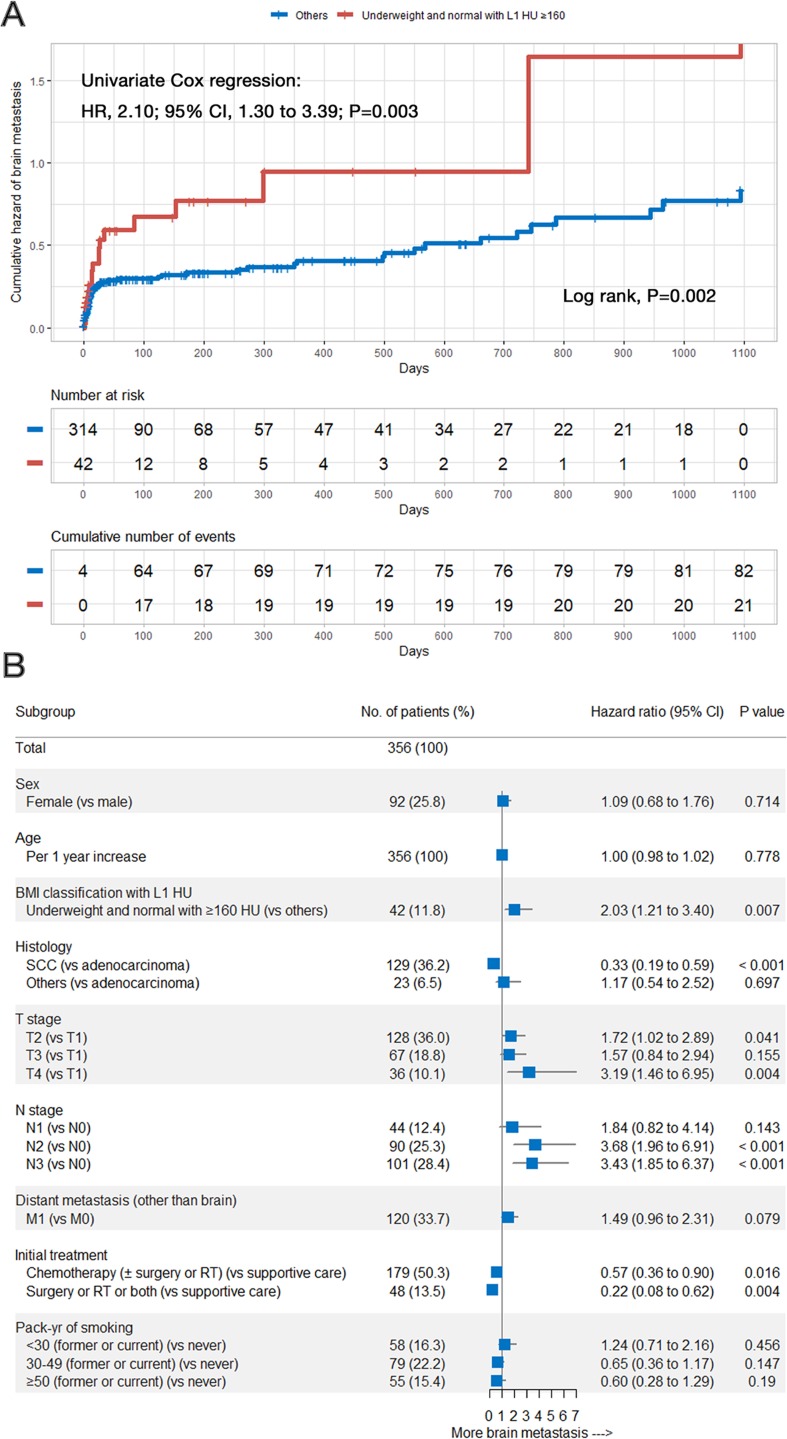
Cumulative hazard curve and forest plot of brain metastasis. (A) Cumulative hazard of brain metastasis in the low BMI with high BMD group and others; (B) Forest plot of estimates from the multivariate Cox regression analysis for predicting brain metastasis according to the predictive factors (adjusted for sex, age [continuous variable], BMI L1 HU classification, histology, T stage, N stage, distant metastasis [other than in the brain], initial treatment, and smoking history in pack-years). BMI = body mass index; BMD = bone mineral density; HU = Hounsfield unit; HR = hazards ratio; CI = confidence interval; SCC = squamous cell carcinoma; RT = radiotherapy.

Most brain metastases occurred rapidly in the early period after lung cancer diagnosis. The incidence of brain metastasis was higher among patients who were underweight or had lower BMI (underweight and normal) (Fig 2B and 2C, *P* = 0.015; *P* = 0.004, respectively). Patients with higher L1 HU (≥160 HU) also tended to have higher rate of brain metastasis in the clinical course of NSCLC (Fig 2D, *P* = 0.055). When the patients were divided into the lower BMI (underweight and normal) with higher BMD (L1 HU ≥160) group and others, the rate of brain metastasis was significantly higher in the lower BMI with higher BMD group than that in the others (Fig 3A, HR, 2.10; 95% CI, 1.30 to 3.39; *P* = 0.003).

### BMI and BMD as independent predictors for brain metastasis in NSCLC patients

The HRs with 95% CIs of the study variables are shown in S2 Table. We found that lower BMI and higher BMD were the independent predictors of brain metastasis in patients with NSCLC (HR, 1.77; 95% CI, 1.16 to 2.70; *P* = 0.009; HR, 1.61; 95% CI, 1.01 to 2.57; *P* = 0.046, respectively). Lower BMI with higher BMD was a stronger independent predictive factor for brain metastasis relative to each lower BMI and higher BMD group (HR, 2.03; 95% CI, 1.21 to 3.40; *P* = 0.007; Fig 3B).

### Paradoxical association between BMI and BMD in NSCLC patients with brain metastasis

**Fig 4:**
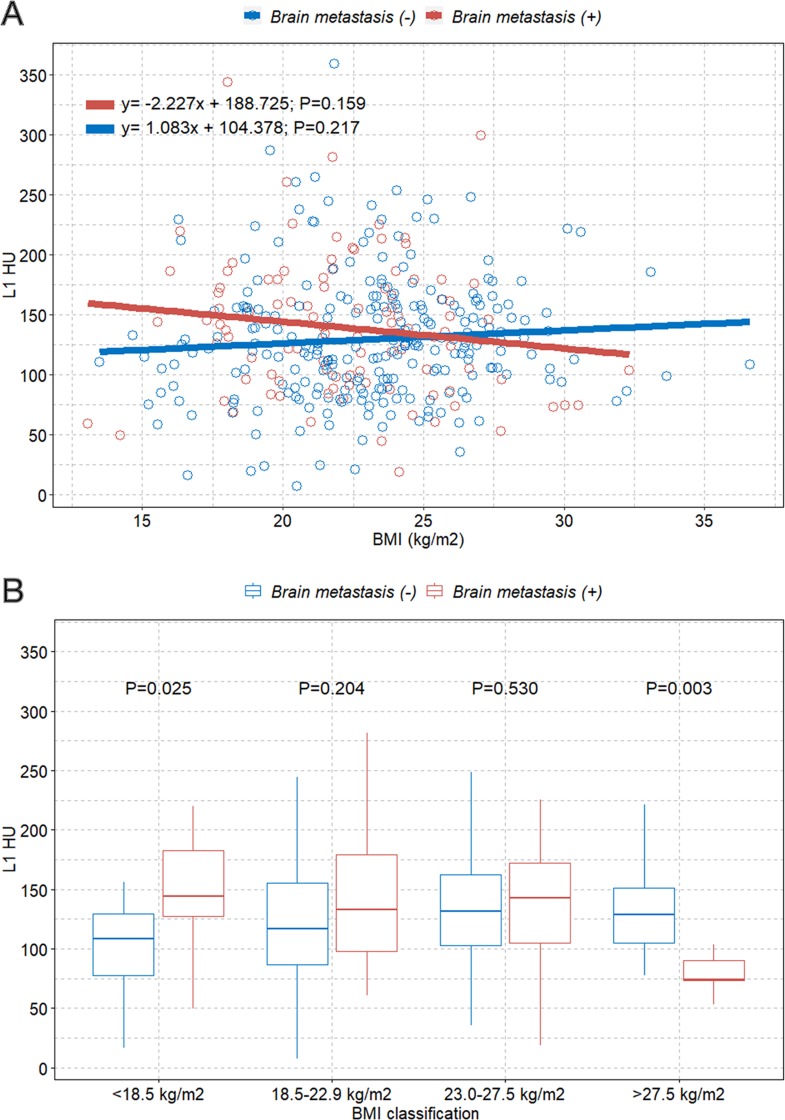
Scatter plot with the linear regression line and box plot. (A) Linear regression line showing the association between BMI and L1 HU values based on the presence of brain metastasis; (B) Boxplots of L1 HU values classified according to four BMI categories (underweight, normal, overweight, and obese). BMI = body mass index; HU = Hounsfield unit.

We observed an overall tendency of negative correlation between BMI and L1 HU value among patients with brain metastasis (B, -2.227; *P* = 0.159) (Fig 4A).

The boxplot also showed a tendency of higher L1 HU values in those with lower BMI and lower L1 HU values in those with higher BMI among patients with brain metastasis patients than those in patients without brain metastasis (Fig 4B). Multivariable linear regression analysis of L1 HU showed an independent negative correlation between BMI and L1 HU among patients with brain metastasis; about 3.3 HU decrease per 1 BMI increase (B, -3.343; 95% CI, -6.352 to -0.333; *P* = 0.030; Table 2).

**Table 2:** Multivariable linear regression analysis of L1 HU value according to predictive factors for brain metastasis in patients with NSCLC.

	Multivariable linear regression analysis			
	Brain metastasis (-)		Brain metastasis (+)	
Variable	β (95% CI)	*P* value	β (95% CI)	*P* value
Sex				
Female (vs male)	-16.751 (-33.650 to 0.149)	0.052	-4.939 (-29.881 to 20.003)	0.695
Age (per 1-year increase)	-2.062 (-2.715 to -1.410)	<0.001	-2.441 (-3.419 to -1.463)	<0.001
BMI (per 1 BMI increase)	0.280 (-1.353 to 1.912)	0.736	-3.343 (-6.352 to -0.333)	0.030
Histology				
SCC (vs adenocarcinoma)	-10.375 (-24.547 to 3.798)	0.151	3.384 (-25.953 to 32.722)	0.819
Others (vs adenocarcinoma)	-5.121 (-33.707 to 23.466)	0.724	0.400 (-37.804 to 38.603)	0.983
T stage (per 1 stage increase)	-0.374 (-6.924 to 6.175)	0.910	-1.719 (-13.227 to 9.789)	0.767
N stage (per 1 stage increase)	4.054 (-1.387 to 9.494)	0.143	11.618 (1.419 to 21.818)	0.026
M1 (vs M0)	-2.770 (-16.075 to 10.535)	0.682	7.897 (-13.593 to 29.388)	0.467
Initial treatment				
Chemotherapy (± surgery or RT) (vs supportive care)	-1.602 (-15.233 to 12.030)	0.817	6.193 (-17.272 to 29.658)	0.601
Surgery or RT or both (vs supportive care)	13.346 (-5.247 to 31.939)	0.159	1.322 (-54.249 to 56.892)	0.962
Smoking history in pack-years				
<30 (vs never)	4.929 (-14.258 to 24.117)	0.613	7.845 (-21.003 to 36.692)	0.590
30–49 (vs never)	3.459 (-13.295 to 20.213)	0.685	-11.377 (-43.254 to 20.501)	0.480
≥50 (vs never)	-9.353 (-26.835 to 8.129)	0.293	-0.645 (-43.263 to 41.972)	0.976

HU, Hounsfield unit; NSCLC, non–small cell lung cancer; CI, confidence interval; BMI, body mass index; SCC, squamous cell carcinoma; RT, radiotherapy

Additionally, N stage showed an independent positive association with L1 HU among patients with brain metastasis (B, 11.618; 95% CI, 1.419 to 21.818; *P* = 0.026). As expected, age also showed a significant negative correlation with L1 HU among both patients with and without brain metastasis. The linear regression analysis between age and BMI and L1 HU is presented in S1 Fig.

### Other predictive factors for brain metastasis

Adenocarcinoma, higher T and N stage, and receiving supportive care showed higher brain metastasis and were independent predictors for brain metastasis in NSCLC patients (S2 Fig.; S2 Table). Patients receiving supportive care showed higher rate of brain metastasis, and this finding may be affected by the patient’s choice of palliative care rather than aggressive treatment due to decreased KPS scores or hopeless situations due to advanced cancer stage at diagnosis. However, the cumulative hazard of brain metastasis in who patients underwent chemotherapy reached a similar level with that of patients receiving supportive care only after more than 2 years.

S3 and S4 Tables show the distribution of patients based on sex, age, and known predictive factors for brain metastasis according to initial BMI and L1 HU classification. There were no significant differences in predictive factors for brain metastasis such as histological type, initial tumor stage, and treatment according to BMI and L1 HU classification at lung cancer diagnosis.

## Discussion

Low BMI and high BMD showed an approximately 1.8- and 1.6-fold increased risk of brain metastasis in patients with NSCLC, respectively, after adjusting for other predictive factors. We also found that patients with low BMI and high BMD simultaneously showed an approximately 2-fold increased risk of brain metastasis. In addition, BMI was negatively correlated with BMD among patients with brain metastasis after adjusting for all other variables. To the best of our knowledge, this study is the first to suggest a possible connection between brain metastasis and low BMI with high BMD in NSCLC patients.

Initially, we hypothesized that low weight and osteoporotic conditions may be related to brain metastasis in patients with lung cancer. Therefore, we initially aimed to analyze the association between low BMI and BMD and presence of brain metastasis in NSCLC patients. However, unexpected results prompted us to investigate reasonable explanations for the observations in the study.

The Wnt/β-catenin signaling pathway is critically involved in both embryonic development and normal adult homeostasis and has also been widely reported to be strongly associated with various cancers [23,24], particularly lung cancer. The Wnt pathway activity is associated with the maintenance of proliferative potential and aggressiveness in NSCLC [25,26]. Previous studies reported that hyperactivation of Wnt/β-catenin signaling promotes brain metastasis from lung cancer [27–29]. In addition, an increase in Wnt signaling activity is also related to metastasis, including brain metastasis from breast cancer and malignant melanoma [30–32]. Therefore, we hypothesized that brain metastasis may be more closely related to the hyperactivation of the Wnt/β-catenin signaling in NSCLC. Previous studies described the differential activity of Wnt pathway in cancer [33–35]. It is generally accepted that activation of Wnt signaling alters the mesenchymal stem cell fate from adipocytes to osteoblasts, and this inhibits adipogenesis and stimulates osteoblastogenesis [36–40]. Osteoblasts and adipocytes are derived from the same precursor bone marrow mesenchymal stem cells [41]. During bone formation, activation of the Wnt/β-catenin signaling represses differentiation of mesenchymal stem cells into the adipocytic lineage. In addition, the peroxisome-proliferator-activated receptor-γ (PPAR-γ), a nuclear receptor and transcription factor, is also regarded as the master moderator of adipogenesis and osteogenesis, similar to Wnt/β-catenin signaling [42]. Suppression of CCAAT/enhancer-binding protein α and PPAR-γ by activation of Wnt signaling is reported to stimulate osteoblastogenesis and represses adipogenesis [43]. Collectively, we hypothesized that hyperactivation of Wnt/β-catenin signaling with subsequent suppression of the PPAR-γ with unclear reason in NSCLC patients may cause lower fat and higher bone mass with higher risk of brain metastasis as shown in our findings. This process subsequently resulted in a paradoxical negative relationship between BMI and BMD in the patients with brain metastasis. This is supported by the positive correlation of weight and BMI with BMD [14,44,45]. However, the exact underlying mechanism for the correlation between brain metastasis and Wnt signaling is unclear. Nguyen et al. reported that hyperactivation of Wnt signaling in lung adenocarcinoma can stimulate brain metastasis independently of changes in intrinsic tumor cell proliferation [27]. They indicated that lung adenocarcinomas with hyperactive Wnt pathway are typically competent to metastasize rapidly in early stage lung adenocarcinomas, unlike breast or prostate cancer metastasis. Our study patients also showed rapid brain metastasis in the early clinical course of NSCLC, and this is thought to be somewhat influenced by the hyperactivation of Wnt signaling.

A recent review reported additional potential biomarkers that are associated with brain metastasis from lung cancer, including the epidermal growth factor receptor, KRAS, anaplastic lymphoma kinase rearrangements, single-nucleotide polymorphisms, miroRNAs, and others [46]. Additionally, E-cadherin and PPAR-γ have also been reported to be associated with brain metastasis in patients with lung cancer [47,48].

Recent meta-studies consistently indicated that patients with high BMI have a lower risk of lung cancer incidence and mortality than those with low BMI [9,11,49,50]. The possible explanations included smoking, cotinine, protective effect on respiratory disease, nutrition, skeletal muscle wasting, time of its determination, and medication [11,12,49]. Increased levels of proinflammatory interleukin 6 have been associated with sarcopenia, low BMI, and the promotion of tumorigenesis and distant metastases [51–53]. In addition, BMI shows an inverse relationship with fatty acid synthase expression, which is a known oncogene. A previous study found that fatty acid synthase is significantly downregulated in obese patients with renal cell carcinoma and is associated with favorable survival outcomes [54]. Furthermore, the growth and proliferation of tumors with high malignant potential require higher energy demands [55]. Further, higher energy demands may naturally lead to the elevation of adipose tissue lipolysis and subsequently result in low BMI in patients with advanced cancers.

Our study has several limitations. First, due to its retrospective nature, the time at which follow-up brain MRI was performed was highly heterogeneous. However, we only included patients who underwent contrast brain MRI within 3 months of diagnosis. Therefore, the presence of brain metastasis was initially evaluated in all patients. Second, BMI may not accurately reflect body fat mass in the study patients. In addition, data of premorbid BMI or initial weight loss due to the cancer were not available. Third, because a BMD test is typically not required in patients with lung cancer, the actual T-score was not available. However, a previous study showed that the L1 HU value showed a relatively reflected the actual BMD, and the cut-off value of 160 HU had a specificity and sensitivity of >70% for distinguishing normal bone density from osteopenia and osteoporosis [13]. Fourth, our proposed mechanisms are hypothetical. However, we believe that the metastatic, aggressive behavior of NSCLC in our study is somewhat associated with Wnt signaling because, to the best of our knowledge, our findings are hard to be explained by other etiologies.

In conclusion, our study showed a significant association between brain metastasis and lower BMI with higher BMD in NSCLC patients. Although hypothetical, considering our findings, we propose a possible connection between brain metastases and Wnt signaling activation in lung cancer. These results may be helpful for predicting brain metastasis during the clinical course of NSCLC, and we expect that they may be helpful to enhance understanding of the underlying mechanism between brain metastasis and lung cancer in the future.

## Supporting information

S1 Fig Scatterplot with linear regression line between age and BMI and L1 HU values in the study patients. (A) Linear regression line showing the association between age and BMI; (B) Linear regression line showing the association between age and L1 HU values. BMI = body mass index; HU = Hounsfield unit.
(TIF)

S2 Fig Cumulative hazard of brain metastasis according to predictive factors. (A) histology; (B) T stage; (C) N stage; (D) initial treatment. SCC = squamous cell carcinoma; RT = radiotherapy.
(TIF)

S1 Table Information on brain metastasis in the study cohort. (DOCX)

S2 Table Uni- and multivariate Cox regression analysis of brain metastasis in patients with NSCLC based on predictive factors. (DOCX)

S3 Table Descriptive statistics of the study patients classified by BMI classification based on known predictors for brain metastasis. (DOCX)

S4 Table Descriptive statistics of the study patients classified by L1 HU value of 160 based on known predictors for brain metastasis. (DOCX)
