# Supplementary figures and images for: Paradoxical relationship between body mass index and bone mineral density in patients with non–small cell lung cancer with brain metastasis
Source: PLoS One. 2019 Jun 21;14(6):e0218825. doi: 10.1371/journal.pone.0218825 (PMC6588256; doi:10.1371/journal.pone.0218825)

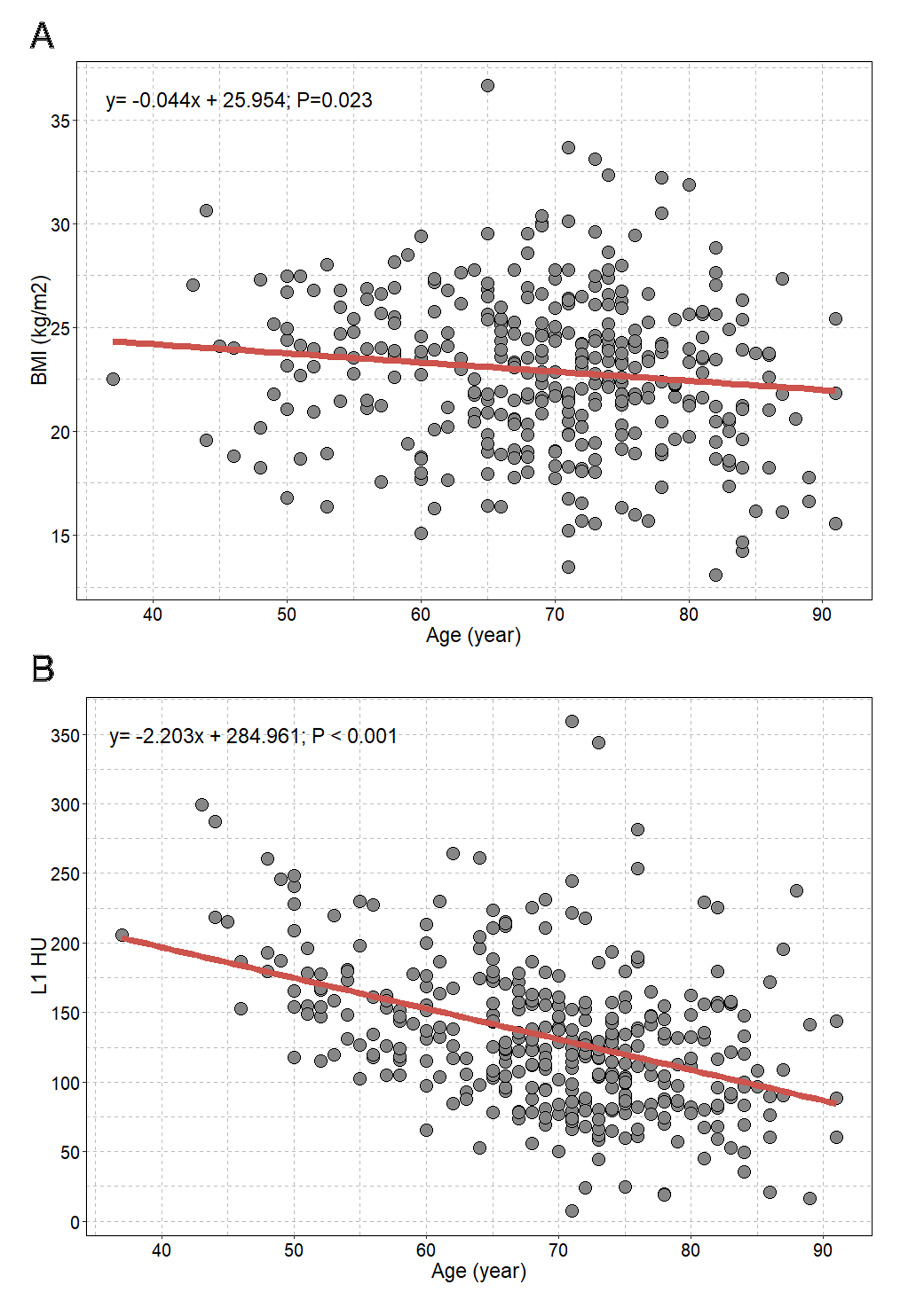

Supplement: S1 Fig — (A) Linear regression line showing the association between age and BMI; (B) Linear regression line showing the association between age and L1 HU values. BMI = body mass index; HU = Hounsfield unit. (TIF) [file pone.0218825.s001.tif]

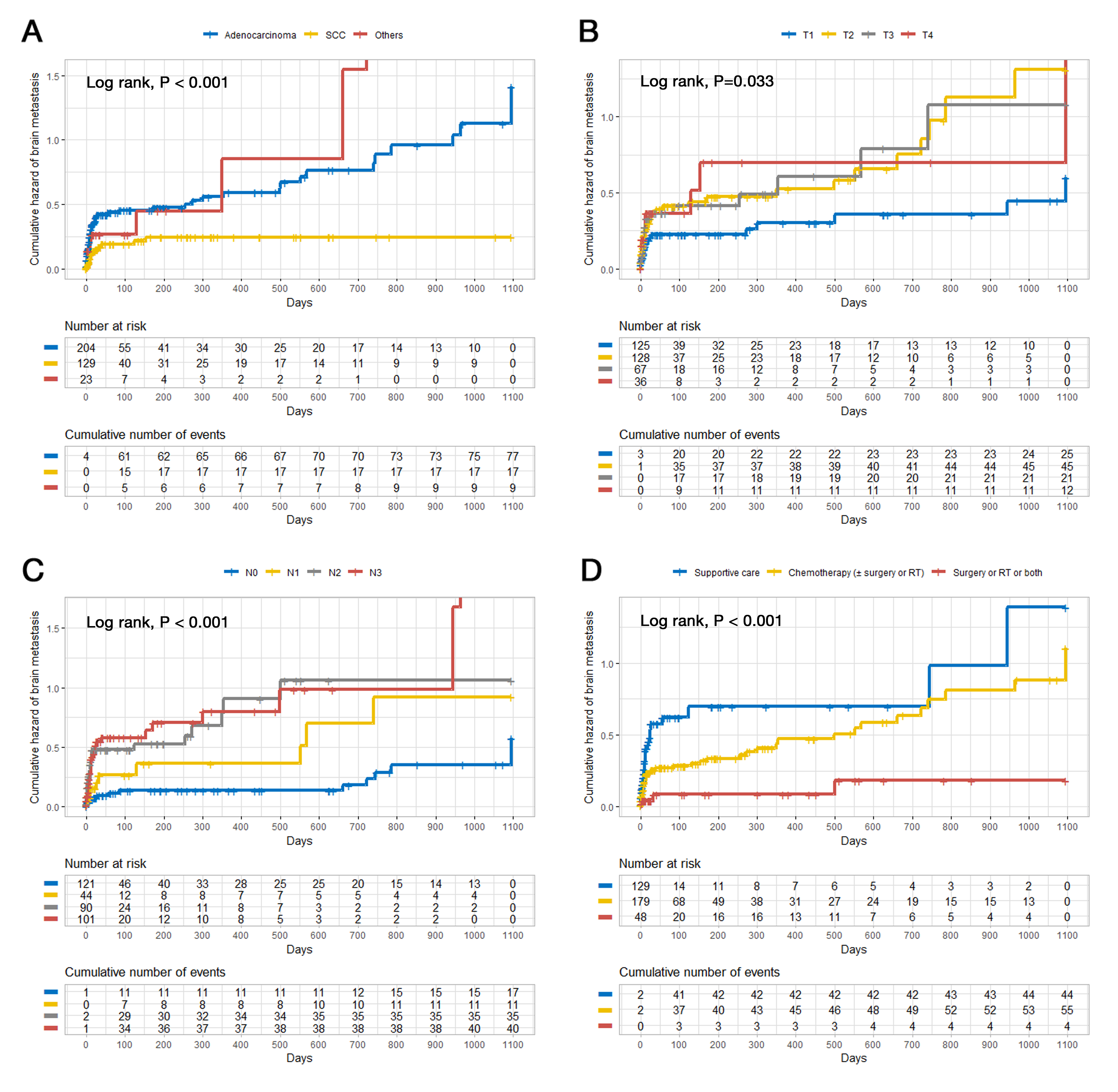

Supplement: S2 Fig — (A) histology; (B) T stage; (C) N stage; (D) initial treatment. SCC = squamous cell carcinoma; RT = radiotherapy. (TIF) [file pone.0218825.s002.tif]
