# Supplementary material for: Paradoxical relationship between body mass index and bone mineral density in patients with non–small cell lung cancer with brain metastasis
Source: PLoS One. 2019 Jun 21;14(6):e0218825. doi: 10.1371/journal.pone.0218825 (PMC6588256; doi:10.1371/journal.pone.0218825)
Supplement: S1 Table — (DOCX) [file pone.0218825.s003.docx]

| Characteristics | Number (%) |
| --- | --- |
| Total patients | 103 (100) |
| Number of brain metastasis |  |
| 1 | 27 (26.2) |
| 2 | 19 (18.4) |
| ≥ 3 | 49 (47.6) |
| Leptomeningeal metastasis | 8 (7.8) |
| Brain metastasis laterality |  |
| Right | 18 (17.5) |
| Left | 16 (15.5) |
| Bilateral or unknown | 69 (67.0) |
| Location of brain metastasis |  |
| Frontal lobe | 53 (51.5) |
| Parietal lobe | 52 (50.5) |
| Temporal lobe | 20 (19.4) |
| Occipital lobe | 24 (23.3) |
| Cerebellum | 34 (33.0) |
| Basal ganglia | 5 (4.9) |
| Brain stem | 10 (9.7) |
| Cerebral unspecified or others | 23 (22.3) |
