# Supplementary material for: Paradoxical relationship between body mass index and bone mineral density in patients with non–small cell lung cancer with brain metastasis
Source: PLoS One. 2019 Jun 21;14(6):e0218825. doi: 10.1371/journal.pone.0218825 (PMC6588256; doi:10.1371/journal.pone.0218825)
Supplement: S2 Table — (DOCX) [file pone.0218825.s004.docx]

|  | Univariate analysis | | Multivariate analysis | |
| --- | --- | --- | --- | --- |
| Variable | HR (95% CI) | P value | HR (95% CI) | P value |
| Sex |  |  |  |  |
| Male | Reference |  | Reference |  |
| Female | 1.57 (1.05 to 2.36) | 0.028 | 1.15 (0.72 to 1.84) | 0.549 |
| Age  (per 1-year increase) | 1.00 (0.98 to 1.02) | 0.824 | 1.00 (0.98 to 1.02) | 0.850 |
| BMI classification |  |  |  |  |
| Underweight and normal | 1.76 (1.19 to 2.61) | 0.005 | 1.77 (1.16 to 2.70) | 0.009 |
| Overweight and obese | Reference |  | Reference |  |
| L1 HU |  |  |  |  |
| <160 | Reference |  | Reference |  |
| ≥160 | 1.49 (0.99 to 2.24) | 0.059 | 1.61 (1.01 to 2.57) | 0.046 |
| Histology |  |  |  |  |
| Adenocarcinoma | Reference |  | Reference |  |
| SCC | 0.33 (0.19 to 0.55) | < 0.001 | 0.33 (0.19 to 0.59) | < 0.001 |
| Others | 1.07 (0.53 to 2.13) | 0.855 | 1.03 (0.47 to 2.26) | 0.946 |
| T stage |  |  |  |  |
| T1 | Reference |  | Reference |  |
| T2 | 1.88 (1.15 to 3.07) | 0.012 | 1.85 (1.10 to 3.13) | 0.021 |
| T3 | 1.80 (1.01 to 3.22) | 0.048 | 1.56 (0.83 to 2.93) | 0.171 |
| T4 | 2.27 (1.13 to 4.52) | 0.020 | 3.25 (1.48 to 7.13) | 0.003 |
| N stage |  |  |  |  |
| N0 | Reference |  | Reference |  |
| N1 | 2.09 (0.98 to 4.47) | 0.057 | 2.11 (0.94 to 4.76) | 0.071 |
| N2 | 4.64 (2.57 to 8.39) | < 0.001 | 3.87 (2.05 to 7.31) | < 0.001 |
| N3 | 4.76 (2.65 to 8.54) | < 0.001 | 3.59 (1.92 to 6.71) | < 0.001 |
| Distant metastasis (other than in the brain) |  |  |  |  |
| M0 | Reference |  | Reference |  |
| M1 | 1.76 (1.18 to 2.61) | 0.005 | 1.46 (0.94 to 2.27) | 0.093 |
| Initial treatment |  |  |  |  |
| Supportive care | Reference |  | Reference |  |
| Chemotherapy (± surgery or RT) | 0.56 (0.38 to 0.85) | 0.006 | 0.54 (0.34 to 0.85) | 0.008 |
| Surgery or RT or both | 0.13 (0.05 to 0.38) | < 0.001 | 0.23 (0.08 to 0.67) | 0.007 |
| Smoking history in pack-years |  |  |  |  |
| Never | Reference |  | Reference |  |
| <30 | 1.85 (0.91 to 3.75) | 0.090 | 1.27 (0.72 to 2.22) | 0.410 |
| 30–49 | 2.50 (1.14 to 5.47) | 0.022 | 0.60 (0.33 to 1.08) | 0.090 |
| ≥50 | 1.65 (0.76 to 3.61) | 0.207 | 0.66 (0.31 to 1.44) | 0.297 |

Abbreviations: NSCLC, non–small cell lung cancer; HR, odds ratio; CI, confidence interval; BMI, body mass index; HU, Hounsfield unit; SCC, squamous cell carcinoma; RT, radiotherapy
