# Supplementary material for: Paradoxical relationship between body mass index and bone mineral density in patients with non–small cell lung cancer with brain metastasis
Source: PLoS One. 2019 Jun 21;14(6):e0218825. doi: 10.1371/journal.pone.0218825 (PMC6588256; doi:10.1371/journal.pone.0218825)
Supplement: S3 Table — (DOCX) [file pone.0218825.s005.docx]

| Variable | Underweight,  <18.5 (n=43) | Normal,  18.5–22.9  (n=131) | Overweight,  23.0–27.5  (n=151) | Obese,  >27.5  (n=31) | P value |
| --- | --- | --- | --- | --- | --- |
| Female patients, n (%) | 11 (25.6) | 29 (22.1) | 42 (27.8) | 10 (32.3) | 0.591 |
| Age (years), mean ± SD | 71.9 ± 10.9 | 70.2 ± 9.9 | 67.9 ± 10.4 | 69.1 ± 8.4 | 0.087 |
| L1 HU, n (%) |  |  |  |  | 0.287 |
| <160 | 34 (79.1) | 98 (74.8) | 108 (71.5) | 27 (87.1) |  |
| ≥160 | 9 (20.9) | 33 (25.2) | 43 (28.5) | 4 (12.9) |  |
| Histology, n (%) |  |  |  |  | 0.336 |
| Adenocarcinoma | 24 (55.8) | 74 (56.5) | 83 (55.0) | 23 (74.2) |  |
| Squamous cell carcinoma | 15 (34.9) | 46 (35.1) | 60 (39.7) | 8 (25.8) |  |
| Others | 4 (9.3) | 11 (8.4) | 8 (5.3) | 0 (0.0) |  |
| T stage, n (%) |  |  |  |  | 0.140 |
| T1 | 13 (30.2) | 48 (36.6) | 53 (35.1) | 11 (35.5) |  |
| T2 | 13 (30.2) | 44 (33.6) | 53 (35.1) | 18 (58.1) |  |
| T3 | 10 (23.3) | 24 (18.3) | 31 (20.5) | 2 (6.5) |  |
| T4 | 7 (16.3) | 15 (11.5) | 14 (9.3) | 0 (0.0) |  |
| N stage, n (%) |  |  |  |  | 0.260 |
| N0 | 10 (23.3) | 46 (35.1) | 54 (35.8) | 11 (35.5) |  |
| N1 | 7 (16.3) | 14 (10.7) | 22 (14.6) | 1 (3.2) |  |
| N2 | 16 (37.2) | 33 (25.2) | 30 (19.9) | 11 (35.5) |  |
| N3 | 10 (23.3) | 38 (29.0) | 45 (29.8) | 8 (25.8) |  |
| Distant metastasis  (other than in the brain), n (%) |  |  |  |  | 0.612 |
| M0 | 27 (62.8) | 83 (63.4) | 106 (70.2) | 20 (64.5) |  |
| M1 | 16 (37.2) | 48 (36.6) | 45 (29.8) | 11 (35.5) |  |
| Initial treatment, n (%) |  |  |  |  | 0.526 |
| Supportive care | 20 (46.5) | 51 (38.9) | 48 (31.8) | 10 (32.3) |  |
| Chemotherapy  (± surgery or RT) | 19 (44.2) | 65 (49.6) | 80 (53.0) | 15 (48.4) |  |
| Surgery or RT or both | 4 (9.3) | 15 (11.5) | 23 (15.2) | 6 (19.4) |  |

BMI, body mass index; HU, Hounsfield unit; RT, radiotherapy
