# Supplementary material for: Paradoxical relationship between body mass index and bone mineral density in patients with non–small cell lung cancer with brain metastasis
Source: PLoS One. 2019 Jun 21;14(6):e0218825. doi: 10.1371/journal.pone.0218825 (PMC6588256; doi:10.1371/journal.pone.0218825)
Supplement: S4 Table — (DOCX) [file pone.0218825.s006.docx]

| Variable | L1 HU <160 | L1 HU ≥160 | P |
| --- | --- | --- | --- |
| Female patients, n (%) | 73 (27.3) | 19 (21.3) | 0.328 |
| Age, mean ± SD, y | 71.5 ± 8.8 | 62.8 ± 11.1 | < 0.001 |
| Histology, n (%) |  |  | 0.924 |
| Adenocarcinoma | 152 (56.9) | 52 (58.4) |  |
| Squamous cell carcinoma | 97 (36.3) | 32 (36.0) |  |
| Others | 18 (6.7) | 5 (5.6) |  |
| T stage, n (%) |  |  | 0.366 |
| T1 | 97 (36.3) | 28 (31.5) |  |
| T2 | 99 (37.1) | 29 (32.6) |  |
| T3 | 45 (16.9) | 22 (24.7) |  |
| T4 | 26 (9.7) | 10 (11.2) |  |
| N stage, n (%) |  |  | 0.186 |
| N0 | 99 (37.1) | 22 (24.7) |  |
| N1 | 31 (11.6) | 13 (14.6) |  |
| N2 | 66 (24.7) | 24 (27.0) |  |
| N3 | 71 (26.6) | 30 (33.7) |  |
| Distant metastasis  (other than in the brain), n (%) |  |  | 0.897 |
| M0 | 176 (65.9) | 60 (67.4) |  |
| M1 | 91 (34.1) | 29 (32.6) |  |
| Initial treatment, n (%) |  |  | 0.551 |
| Supportive care | 99 (37.1) | 30 (33.7) |  |
| Chemotherapy  (± surgery or RT) | 130 (48.7) | 49 (55.1) |  |
| Surgery or RT or both | 38 (14.2) | 10 (11.2) |  |

HU, Hounsfield unit; RT, radiotherapy
